# Supplementary material for: Dandelion Leaf Aqueous Extract Relieves Hyperuricemia and Its Complications via Modulating Uric Acid Metabolism, Renal Inflammation, and Gut Microbes
Source: Foods. 2025 Nov 10;14(22):3843. doi: 10.3390/foods14223843 (PMC12651310; doi:10.3390/foods14223843)
Supplement: Supplementary file 1 [file foods-14-03843-s001.zip › foods-3942006-supplementary.pdf]

Supporting Information

Table S1. The gradient elution program of the mobile phase

Time (min)	Flow Rate (mL/min)	A%
0-1	0.3	2
1-5	0.3	20
5-10	0.3	50
10-15	0.3	80
15-20	0.3	95
20-27	0.3	95
27-28	0.3	2
28-30	0.3	2

Table S2 Primer sequences for RT-qPCR

Description	Sense primer (5′ → 3′)	Antisense primer (5′ → 3′)
β-Actin	GTGACGTTGACATCCGTAAAGA	GTAACAGTCCGCCTAGAAGCAC
OAT1	GCCTTGATGGCTGGGTCTATG	AGCCAAAGACATGCCCGAGA
OAT2	CAACTGCGGAATCTGGTGCT	ATCAGGCAGGGCACAATGATG
GLUT9	GATGCTCATTGTGGGACGGTT	CTGGACCAAGGCAGGGACAA
ABCG2	GGCCTGGACAAAGTAGCAGA	GTTGTGGGCTCATCCAGGAA
IL-1β	GCAACTGTTCCTGAACTCAACT	ATCTTTTGGGGTCCGTCAACT
Caspase-1	TATCCAGGAGGGAATATGTG	ACAACACCACTCCTTGTTTC
IL-6	CTGCAAGAGACTTCCATCCAG	AGTGGTATAGACAGGTCTGTTGG
NLRP3	GTGGTGACCCTCTGTGAGGT	TCTTCCTGGAGCGCTTCTAA
TNF-α	CAGGCGGTGCCTATGTCTC	CGATCACCCCGAAGTTCAGTAG
TLR-4	GGAAGACAAAAGAAAGACAGCCC	TGGGGAGATTCTTGATCTGCT
MyD88	TCATGTTCTCCATACCCTTGGT	AAACTGCGAGTGGGGTCAG
NF-κB	ATGGCAGACGATGATCCCTAC	TGTTGACAGTGGTATTTCTGGTG

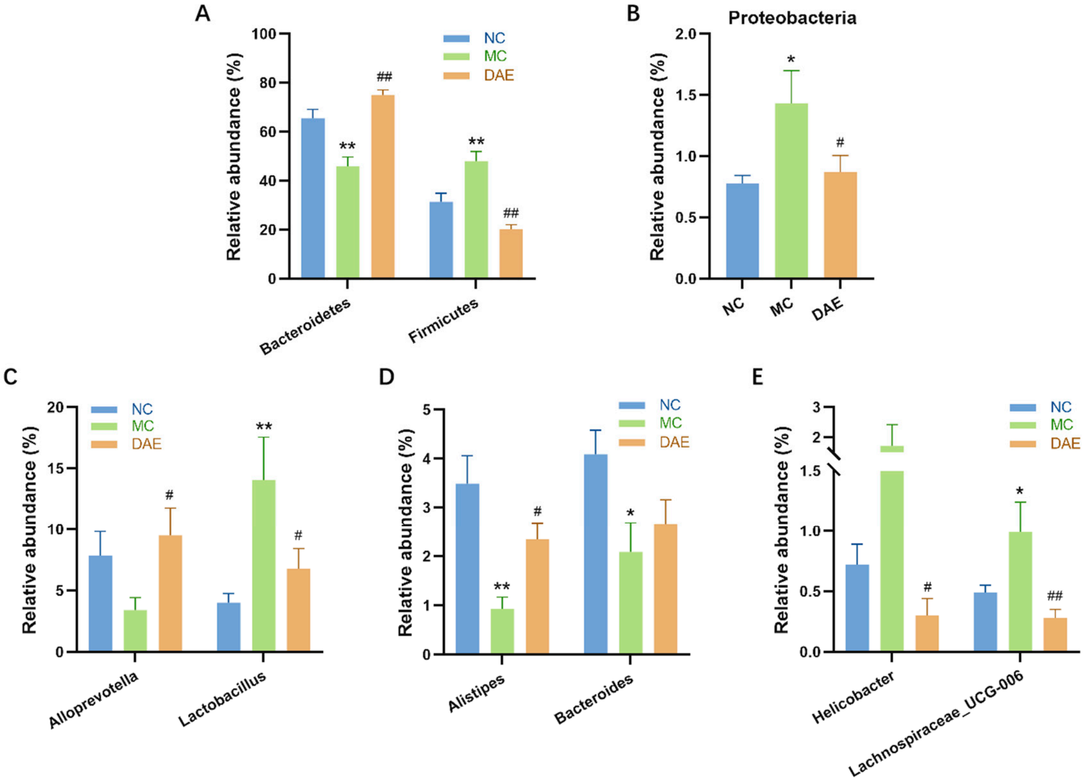

Figure S1 Changes of gut microbiota at the phylum and genus levels. Relative abundance of Bacteroidetes, Firmicutes (A), and Proteobacteria (B). Relative abundance of *Lactobacillus*, *Alloprevotella* (C), *Alistipes*, *Bacteroides* (D), *Helicobacter* and *Lachnospiraceae UCG-006* (E). * $p < 0.05$ and ** $p < 0.01$: NC group vs MC group; # $p < 0.05$ and ## $p < 0.01$: MC group vs DAE group.
